# Do modeling liquid and glycerin gel compromise the color stability of one-shade composites

**DOI:** 10.1007/s10266-025-01301-3

**Published:** 2026-01-06

**Authors:** Ezgi Erden Kayalidere, Merve Sahin

**Affiliations:** Department of Restorative Dentistry, Faculty of Dentistry, Istanbul Kent University, Beyoglu, 34433 Istanbul, Turkey

**Keywords:** CIEDE2000, Color change, Glycerin gel, One-shade composites, Modeling liquid

## Abstract

This study aims to investigate the effect of modeling liquid and glycerin gel on the color stability of one-shade composites. 120 specimens were prepared using four one-shade resin composites, one conventional composite, one modeling liquid, and one glycerin gel. The specimens were divided into four groups: Group 1, only polished; Group 2, modeling liquid applied followed by polishing; Group 3, modeling liquid applied without polishing; and Group 4, modeling liquid and glycerin gel applied without polishing. All specimens were stored in a coffee solution for one week. Color measurements were performed using a spectrophotometer at baseline and after coffee staining. Data were analyzed using the Kruskal–Wallis test and Post Hoc Dunn–Bonferroni test. Clinically unacceptable color differences were obtained in all composite resins after coffee immersion. In Group 1, Omnichroma and Zenchroma Universal exhibited significantly lower ΔE₀₀ values compared to Vittra APS Unique. In Group 2, Zenchroma Universal showed significantly lower ΔE₀₀ values than Charisma Topaz ONE. In Group 3, the ΔE₀₀ value of Charisma Topaz ONE was lower than that of Vittra APS Unique. In Group 4, Zenchroma Universal showed significantly lower values compared to Vittra APS Unique. The susceptibility of composites to discoloration varied based on surface treatment. Material selection should consider not only esthetic and mechanical properties but also long-term color stability. Modeling liquids facilitate composite handling and improve surface adaptation; however, optimal restoration longevity requires proper finishing and polishing. Combining modeling liquid with glycerin gel and Mylar strips without subsequent polishing may increase surface roughness and susceptibility to staining. This can compromise long-term esthetics and plaque resistance. Polishing not only removes residual inhibition layers but also enhances surface smoothness, reducing discoloration risk. Clinicians should integrate polishing into restorative protocols when using surface wetting agents to ensure improved manipulation during placement and optimal esthetic and functional outcomes over time.

## Introduction

Advancements in adhesive technology have made direct composite resin restorations a popular choice for correcting esthetic issues related to tooth shape and color [1, 2]. These restorations are applied using minimally invasive techniques and mimic the optical properties of natural teeth [3]. Their relatively simple placement enables precise anatomical shaping. However, the viscous nature of resin monomers can hinder manipulation and cause sticking to instruments. To address this, various modeling liquids have been developed to enhance compatibility, adaptation, and contouring [4–7]. Applied to instruments or brushes, these liquids help achieve smoother surfaces and facilitate faster finishing and polishing [8, 9]. They may also be used between composite layers or on the final layer [10].

Modeling liquids contain methacrylate compounds without fillers. These include urethane dimethacrylate (UDMA), bisphenol A-glycidyl methacrylate (Bis-GMA), or triethylene glycol dimethacrylate (TEGDMA) [10, 11]. Although modeling liquids share the same methacrylate derivatives as composite resins, structural deterioration may occur over time in composite restorations when instruments are wetted with modeling liquid [10, 12]**.**

Composite resins are materials prone to discoloration when exposed to food and beverages and may also exhibit a reduction in hardness over time. This is related to the oxygen inhibition layer (OIL) formed in the last cured layer as a result of the polymerization reaction of the composite with atmospheric oxygen [13]. Oxygen is a powerful inhibitor that may slow down or halt polymerization. Free radicals react with oxygen, not with monomers, to form stable but unreactive peroxide radicals. As a result of this reaction, polymerization cannot be completed on the composite surface and residual monomers remain. This layer is called OIL [14, 15]. OIL should be eliminated or reduced during light curing because it negatively affects the surface quality of the restoration [16, 17]**.**

Several methods can reduce OIL formation: finishing and polishing process after light curing, using Mylar strips, or the application of a barrier that prevents the surface from coming into contact with oxygen [18, 19]. One of the materials that can be used for this purpose is glycerin [3, 14, 20]. Glycerin (C₃H₈O₃) is a polyhydric alcohol compound (polyol) with three hydroxyl groups in a molecule, or known as trivalent alcohol. Glycerin is a clear, colorless, viscous, odorless, sweet-tasting solution. Pure glycerin is resistant to oxygen in the air [20, 21]. The use of glycerin gel can prevent the formation of an OIL in polymerization by preventing oxygen contact with free radicals on the surface of the composite resin [14, 22].

Although one-shade composites have become increasingly widespread in recent years, findings regarding their color stability compared to conventional multi-shade composites remain inconsistent; some studies have reported greater, others lower, and some similar levels of color change [23–27].

Modeling liquids, while facilitating composite manipulation, may induce undesirable alterations in the composite structure. Similarly, while glycerin has been shown to prevent OIL formation by blocking oxygen contact during light curing, its effect on color stability when applied in combination with modeling liquid has not been extensively studied. Considering the increasing clinical use of one-shade composites, clarifying the potential impact of adjuncts such as modeling liquids and glycerin gel on their long-term color stability is crucial.

Thus, this in vitro study investigates the effect of modeling liquid and glycerin gel application on the color change of one-shade composites. The null hypotheses tested were: (1) Modeling liquid application has no significant effect on the color stability of one-shade composites, (2) Immersion in a coffee solution does not result in clinically unacceptable ΔE_00_ values in one-shade composites, (3) Glycerin gel application over modeling liquid has no significant effect on the color of one-shade composites.

## Materials and methods

### Specimens’ size determination

Specimen size was determined using G*Power Ver. 3.1.5 (Germany), based on an effect size of 0.25 from a previous study [6]. A minimum of 28 specimens per group was required (α = 0.05, power = 0.80). To account for possible specimen loss, 30 specimens were included per group (*n* = 120).

### Preparation of composite specimens

**Fig. 1:**
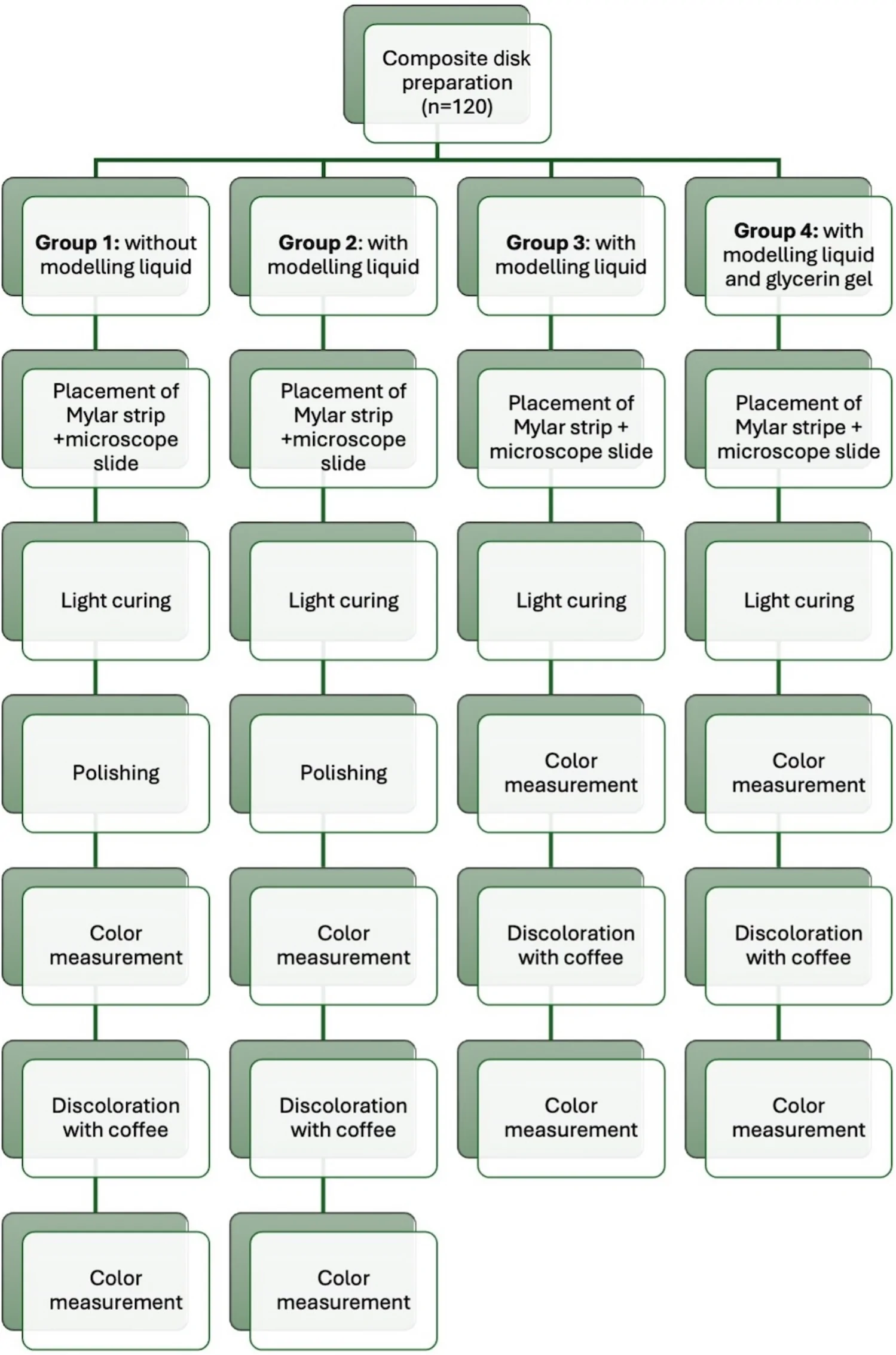
Workflow of composite disk preparation and specimen allocation

This in vitro study evaluated the effect of modeling liquid on the color stability of five different composites against a gray background. A total of 120 specimens were prepared and processed according to the workflow presented in Fig. 1. The composite materials (Omnichroma, Tokuyama; Vittra APS Unique, FGM; Zenchroma Universal, President Dental; Charisma Topaz One, Kulzer; IPS Empress Direct, Ivoclar), modeling liquid (Modeling Liquid, GC Corp.), and glycerin gel (Miraderm) are shown in Table 1.

**Table 1:** Tested resin composites, modeling liquid, and glycerin gel

Materials	Organic Matrix	Inorganic Matrix	Resin Type	Manufacturer
Omnichroma	TEGDMA, UDMA, Mequinol, Dibutyl hydroxyl tolüene, UV absorber	Spherical silica-zirconia filler (mean particle size 0.3 μm, particle size range: 0.2 to 0.4 μm) (68 vol %)	Nanofilled	Tokuyama Dental Corporation, Tokyo, Japan
Charisma Topaz ONE	TCD-Urethaneacrylate, TEGDMA, UDMA	Barium aluminum boro flour silicate glass, silica, titanium dioxid (58 vol %)	Nanohybrid	Kulzer, Hanau, Germany
Zenchroma Universal	Bis-GMA, UDMA, TEMDMA	Glass powder. silicon dioxide (53 vol %)	Microhybrid	President Dental, Allershausen, Germany
Vittra APS Unique	Mixture of methacrylate monomers, photo initiator composition (APS), Other Components: co-initiators, stabilizers and silane	Boron-aluminum- silicate glass. (52–60 vol %)	Nanohybrid	FGM Dental, Joinville, Santa Catarina, Brazil
IPS Empress Direct	UDMA Bis-GMA TEGDMA	Barium glass, Ytterbium trifluoride, mixed oxide, silicon dioxide and copolymer (52–59 vol %)	Nanohybrid	Ivoclar Vivadent, Schaan, Liechtenstein
Modeling Liquid	UDMA 2-Hydroxy-1,3-dimethacryloxypropane, 2-hydroxyethyl methacrylate Other Components: initiator, stabilizer	Silicon dioxide (silane-treated)		GC Corp., Tokyo, Japan
Glycerin gel	Pure glycerin			Miraderm Pharma, Istanbul, Turkey

Note: All information has been provided by the manufacturers; *Bis−GMA* Bisphenol A Glycidyl Methacrylate, *TEGDMA* triethyleneglycol dimethacrylate, *TCD* Tricyclodecane, *TEMDMA* Triethylene Glycol Mono Dimethacrylate, *UDMA* urethane dimethacrylate

A single operator prepared a total of 120 disk-shaped composite specimens (5 mm diameter × 2 mm height) using a Teflon mold. Uncured composite was placed into the mold, and group-specific procedures (application of modeling liquid and/or glycerin gel) were performed on the uncured surface before covering the specimen with a polyester matrix strip**.** Subsequently, all specimens were covered with a polyester matrix strip (Mylar Strip, SS White Co.) and polymerized through a 1 mm thick microscope slide using a light-curing unit (Bluephase PowerCure, Ivoclar Vivadent) with a wavelength range of 390–500 nm for 20 s. All specimens were stored in distilled water at 37 °C for 24 h.

**Fig. 2:**
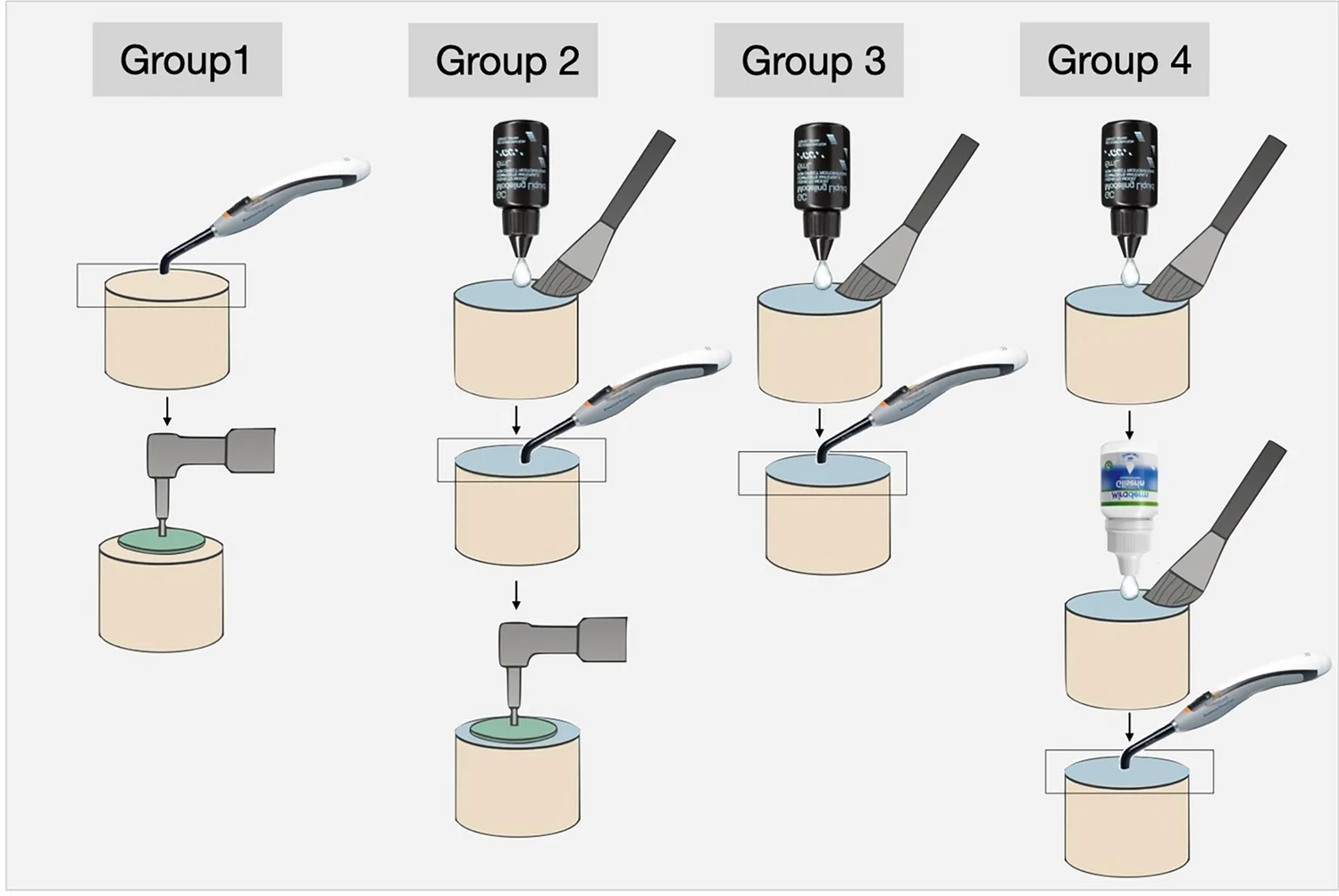
Schematic representation of the group-specific experimental procedures

Each group consisted of 30 specimens, and the specific procedural steps applied to each group are shown in Fig. 2.

**Group 1:** no modeling liquid or glycerin gel was applied to the final increment surface**.** After placing the Mylar strip, specimens were light-cured**,** then polished under dry conditions using Sof-Lex disks (3M ESPE), progressing from coarse to superfine. Coarse and medium disks were used at 10,000 rpm, and fine/superfine at 30,000 rpm, each for 15 s.

**Group 2:** modeling liquid (GC Corp.) was applied to the uncured surface. One drop of modeling liquid was dispensed onto a mixing pad, and a sable brush was lightly touched to the liquid to pick up a minimal amount. Any excess was thoroughly removed using a paper tissue. A single ultrathin, uniform film was applied to each specimen. The brush was not reloaded during the application of a single specimen; between specimens, the brush was cleaned and freshly touched to the modeling liquid to ensure consistent and standardized application. After placing the Mylar strip, the specimens were light-cured, and polishing was performed as described for Group 1.

**Group 3:** modeling liquid was applied to the uncured surface as in Group 2; however, no polishing was performed.

**Group 4:** modeling liquid was applied as in Group 2, followed by the application of glycerin gel (Miraderm) with a separate brush on the uncured composite surface. After placing the Mylar strip, specimens were light-cured. After curing, the glycerin layer was rinsed off with water, and no polishing was performed.

### Simulated staining protocol

Thirty specimens in each group were immersed in coffee solution (Nescafé Classic; Nestlé) for 7 days at 37 °C to simulate staining. According to the manufacturer’s recommendation, 2 g of coffee powder was added to 200 mL of boiling water and filtered after 10 min, then poured into containers. The solution was freshly prepared and changed every day. After each staining period, the specimens were rinsed with distilled water and the surface residues were gently removed with a cotton pellet.

### Color measurements

The color parameters of the specimens were assessed using the Commission Internationale de l’Eclairage (CIE) L*a*b* system with a spectrophotometer (VITA Easy Shade V; VITA Zahnfabrik) against a neutral gray background in two stages. The spectrophotometer was calibrated according to the manufacturer’s recommendations before each measurement. After drying the specimens with tissue paper, three consecutive measurements were taken on each surface, and the average L, a, and b values were calculated. The color change levels in the specimens were calculated using the CIEDE2000 formula [28]:

$$\Delta E_{00} = \sqrt {\left( {\frac{{\Delta L^{\prime } }}{{k_{L} S_{L} }}} \right)^{2} + \left( {\frac{{\Delta C^{{^{\prime } }} }}{{k_{C} S_{C} }}} \right)^{2} + \left( {\frac{{\Delta H^{{^{\prime } }} }}{{k_{H} S_{H} }}} \right)^{2} + R_{T} \left( {\frac{{\Delta C^{{^{\prime } }} }}{{k_{C} S_{C} }}} \right)\left( {\frac{{\Delta H^{{^{\prime } }} }}{{k_{H} S_{H} }}} \right)}$$

ΔL, ΔC, and ΔH represent the differences in lightness, chroma, and hue, respectively, for a pair of points. The weighting functions (SL, SC, SH) adjust how the color difference varies based on the location in the L, a, b coordinates. The parametric factors (kL, kC, kH) are coefficients used to correct for experimental conditions. Under standard conditions, all of these factors are set to 1 (kL = kC = kH = 1). To correct for the interaction between chroma and hue differences, a rotation function (RT) has been added, particularly in the blue color region.

In our study, we referenced the ΔE₀₀ value of 0.8 as the perceptibility threshold (PT) and 1.8 as the acceptability threshold (AT) [29].

### Statistical analysis

NCSS 2007 (Number Cruncher Statistical System, Kaysville) software was used for statistical analyses. The normality of quantitative variables was assessed using the Shapiro–Wilk test and graphical analysis. For comparisons involving three or more groups of non-normally distributed quantitative variables, the Kruskal–Wallis test was applied, followed by the Post Hoc Dunn–Bonferroni test for pairwise comparisons. A *p* value of < 0.05 was considered statistically significant.

## Results

Tables 2 and 3 present the mean ΔE₀₀ values of the resin composites following coffee immersion. The color changes ranged between 8.0 and 21.4 ΔE₀₀ units, and the type of resin composite significantly influenced the degree of color change (*p* < 0.001).

**Table 2:** Comparison of ΔE_00_ values by composite type within each group

Composites	Group 1	Group 2	Group 3	Group 4
Omnichroma (n = 6)	8.42 ± 0.82 (8.3)^A^	11.08 ± 0.74 (10.8)^CD^	13.46 ± 0.77 (13.3)^EF^	17.13 ± 2.19 (16.3)^GH^
Charisma Topaz ONE (n = 6)	9.60 ± 0.91 (9.5)^AB^	14.10 ± 2.18 (13.7)^D^	12.18 ± 1.37 (12.3)^E^	17.65 ± 1.66 (17.3)^GH^
Zenchroma Universal (n = 6)	8.92 ± 1.28 (8.7)^AC^	8.07 ± 1.48 (7.5)^C^	14.49 ± 1.38 (14)^EF^	15.70 ± 1.63 (15.7)^G^
Vittra APS Unique (n = 6)	12.19 ± 1.34 (12.3)^B^	11.61 ± 2.55 (11.1)^CD^	14.75 ± 1.30 (14)^F^	21.44 ± 2.30 (20.9)^H^
IPS Empress Direct (n = 6)	11.12 ± 1.08 (11.3)^BC^	12.75 ± 1.83 (12)^D^	12.43 ± 1.29 (12.5)^EF^	20.95 ± 2.17 (20.1)^H^
*p value*	0.001*	0.001*	0.013*	0.001*

Kruskal Wallis Test, *p<0.05, The same uppercase superscripts in the same column indicate no statistical difference

**Table 3:** Comparison of the ΔE_00_ values of composites between groups

Composites	Group 1	Group 2	Group 3	Group 4	*p *value
Omnichroma (*n* = 24)	8.42 ± 0.82 (8.3)^A^	11.08 ± 0.74 (10.8)^AB^	13.46 ± 0.77 (13.3)^BC^	17.13 ± 2.19 (16.3)^C^	0.001*
Charisma Topaz ONE (*n* = 24)	9.60 ± 0.91 (9.5)^A^	14.10 ± 2.18 (13.7)^BC^	12.18 ± 1.37 (12.3)^AB^	17.65 ± 1.66 (17.3)^C^	0.001*
Zenchroma Universal (*n* = 24)	8.92 ± 1.28 (8.7)^A^	8.07 ± 1.48 (7.5)^A^	14.49 ± 1.38 (14)^B^	15.70 ± 1.63 (15.7)^B^	0,001*
Vittra APS Unique (*n* = 24)	12.19 ± 1.34 (12.3)^A^	11.61 ± 2.55 (11.1)^A^	14.75 ± 1.30 (14)^AB^	21.44 ± 2.30 (20.9)^B^	0.001*
IPS Empress Direct (*n* = 24)	11.12 ± 1.08 (11.3)^A^	12.75 ± 1.83 (12)^A^	12.43 ± 1.29 (12.5)^A^	20.95 ± 2.17 (20.1)^B^	0.002*

Kruskal Wallis Test & Post Hoc Dunn Bonferroni test, *p<0.05, The same uppercase superscripts on the same line indicate no statistical difference

Statistically significant differences in ΔE₀₀ values were observed among the composite types in all groups (*p* < 0.05). In Group 1, Omnichroma exhibited significantly lower ΔE₀₀ values compared to IPS Empress Direct (*p* = 0.035) and Vittra APS Unique (*p* = 0.002). Additionally, Zenchroma Universal demonstrated significantly lower color change than Vittra APS Unique (*p* = 0.006).

In Group 2, Zenchroma Universal showed significantly lower ΔE₀₀ values than both IPS Empress Direct (*p* = 0.021) and Charisma Topaz ONE (*p* = 0.001). In Group 3, the ΔE₀₀ value of Charisma Topaz ONE was lower than that of Vittra APS Unique (*p* = 0.049). In Group 4, Zenchroma Universal again showed significantly lower values compared to IPS Empress Direct (*p* = 0.016) and Vittra APS Unique (*p* = 0.006).

In all the composites, statistically significant differences in ΔE00 values were observed among the groups (*p* < 0.05). Group 4 exhibited significantly higher ΔE₀₀ values than Groups 1 and 2 in Omnichroma, IPS Empress Direct, Zenchroma Universal, and Vittra APS Unique (*p* < 0.05). In Omnichroma and Zenchroma Universal, Group 3 also showed significantly higher values than Group 1. In Charisma Topaz ONE, Group 4 values were significantly higher than those of Groups 1 and 3 (*p* < 0.05), while Group 2 values were also higher than Group 1 (*p* = 0.01). In Vittra APS Unique, only Group 4 showed significantly higher values compared to Groups 1 and 2 (*p* < 0.01).

## Discussion

According to the results of this study, all three null hypotheses were rejected. The application of modeling liquid significantly influenced the color change of one-shade composites, depending on both the surface treatment method and the composite material tested. Similarly, the effect of glycerin gel on color change varied based on the composite material, with greater discoloration observed on surfaces where glycerin was applied. Furthermore, immersion in coffee for one week led to clinically unacceptable color changes in all tested composite materials.

Composite materials and restorative techniques that simplify shade selection in dentistry are highly preferred by dentists. The existence of multiple enamel and dentin shades makes correct shade selection and shade matching difficult. In recent years, "one-shade" composite resins have been developed to speed up the clinical process and reduce the shade diversity of resin composites [3]. Especially in the anterior region composite restorations, one of the most desired features is to obtain minimal ΔE values. However, due to their polymeric structure, composite materials can degrade over time, resulting in the deterioration of their color appearance [3, 4].

In this study, color change after 1 week of coffee immersion was evaluated in five composites with four different surface treatments. Mean ΔE_00_ values ranged from 8.42 to 12.19 in Group 1, 8.07 to 14.10 in Group 2, 12.18 to 14.75 in Group 3, and 15.70 to 21.44 in Group 4. The 50:50 perceptibility (ΔE_00_ = 0.8) and acceptability (ΔE_00_ = 1.8) thresholds established by ISO (2016) were considered [29, 30]. All composites in all groups exceeded the acceptability threshold after coffee staining, consistent with findings in studies on one-shade composites [2, 11]. Some studies have found that one-shade composites exhibit greater color change than conventional composites after exposure to various staining media[23, 27], while other studies have found that these composites exhibit less or similar color change [24, 25]. In our study, when evaluated group by group based on the applied surface treatment, Zenchroma Universal in Groups 1, 2, and 4, Omnichroma and Charisma Topaz ONE in Group 1 showed lower color change than the conventional composite. Staining susceptibility in composite resins is influenced by various factors, including the composition of the resin matrix, the type and particle size of the filler, the efficiency of polymerization, and surface roughness [31]. These factors can play a determining role in the color stability of the composite material by directly affecting its water absorption, permeability to dyes, and surface properties.

Statistically significant differences in ΔE₀₀ values were found among composites across all groups, with the composites showing the highest and lowest color changes varying by group. For instance, Omnichroma exhibited the lowest color change in Group 1, Zenchroma Universal in Groups 2 and 4, and Charisma Topaz ONE in Group 3. These results suggest that color stability depends not only on resin matrix and filler composition but also on surface treatments, surface topography, and overall microstructure**.** In all groups except Group 2, higher ΔE₀₀ values were observed in Vittra APS Unique. Similar to our study, Vittra APS Unique showed higher ΔE₀₀ values after coffee immersion compared to other one-shade composites [26, 32]. Vittra APS Unique is a nanohybrid composite containing Bis-GMA, UDMA, and TEGDMA monomers, leading to relatively high resin matrix hydrophilicity and increased water absorption, which may enhance staining over time. Although pigment additives are not detailed by the manufacturer, studies indicate the presence of organic and inorganic pigments affecting color stability and stain susceptibility [32].

Some studies have reported positive effects of modeling liquid on color stability, while others have found no significant or even negative effects [5, 7, 9]. In this study, no significant difference in ΔE_00_ values was observed between Group 1 (polishing only) and Group 2 (polishing after modeling liquid), except for Charisma Topaz ONE. The fact that the finishing and polishing were applied after the modeling liquid application in Group 2 may have caused the modeling liquid remaining on the surface to be largely removed. Comparing Group 3 (modeling liquid only) to Group 2 showed generally higher ΔE_00_ values in Group 3, though differences were significant only for Zenchroma Universal. Similarly, comparisons between Groups 3 and 1 showed no significant differences except for Omnichroma and Zenchroma Universal. One study found that among seven composites treated with modeling liquid under polyester strips, only Filtek Silorane and Filtek Ultimate showed higher color change [5]. Another study using surface roughness and FE-SEM analyses reported that modeling liquid created smoother, more homogeneous surfaces. The lack of a significant color difference in some composites in Group 3, where only modeling liquid was applied, compared to Groups 1 and 2, may be related to the smoother and more homogeneous surface layer formed by the modeling fluid [11]. Conversely, another study reported that a similar color change was observed in the conventional composite applied with modeling resin in the groups finished only with mylar strip. The study emphasized that the use of modeling resin, particularly in esthetic areas, should be reduced and that these materials can affect the chemical structure of composites [8]. These results suggest that the effect of modeling liquid on color stability depends on application method and material composition. Thus, polishing may still be necessary after using modeling liquid to ensure optimal surface quality and esthetics.

The oxygen inhibition layer was controlled by applying glycerin gel in Group 4, and the effect of this application on color was evaluated together with the modeling liquid. Glycerin was used because it is a water-soluble gel that can be easily removed after performing its oxygen-blocking function during photopolymerization. Thanks to its transparent structure, it does not prevent the light transmittance of polymerization devices, and studies have reported that it does not create negative chemical effects on the surface of resins. Additionally, it is an economical, affordable, and practical material [19, 21].

We also aimed to observe the effect of applying modeling liquid immediately afterward. However, in our study, the ΔE_00_ value of all composites in Group 4 was found to be higher than that of the other groups. Although the ΔE_00_ value is higher than that of other groups, no statistically significant difference was observed in ΔE_00_ values compared to Group 3 (which did not receive glycerin application), except for Charisma Topaz ONE and IPS Empress Direct composites. These results show that the application of glycerin does not have a generally positive effect on color stability. Moreover, there were more visible air bubbles on the surface of the specimens in Group 4 compared to the other groups. This may be attributed to the combined application of modeling liquid, glycerin gel, and a Mylar strip. It has been reported that the use of a Mylar strip can trap air bubbles during placement [33, 34]. The use of a Mylar strip following the application of modeling liquid and glycerin gel may have contributed to the increased number of air bubbles.

In the literature, some studies applied glycerin gel immediately before the polymerization of the composite, while others applied it immediately after polymerization [13, 15, 16, 22]. In our study, applying glycerin gel before light exposure may have contributed to this result. In a study, the use of glycerin increased the conversion rate in the tested composite resins, but did not affect surface staining due to coffee. The lowest ΔE_00_ values were obtained in the finishing and polishing groups [13]. In another study, higher discoloration was observed in specimens polymerized without an oxygen barrier (such as glycerin or a Mylar strip) [17]. Similarly, another study showed lower ΔE values were observed in specimens to which glycerin was applied than in those to which it was not applied [5, 21]. These different results in the literature may be due to the application method of glycerin and the difference in the composite resin used. The difference of our study from others is that glycerin was applied on the modeling liquid and polymerized on the mylar strip.

From a clinical standpoint, the use of modeling liquid should always be followed by polishing to remove potential surface residues. The combination of modeling liquid and glycerin without polishing may compromise esthetics by increasing staining susceptibility.

This study has several limitations that should be considered when interpreting the results. As it was conducted under in vitro conditions, the findings may not fully reflect clinical reality, since dynamic intraoral factors such as saliva flow and temperature changes could not be simulated. Only one type of modeling liquid and a single staining agent (coffee) were used; different results might have been obtained with alternative materials or exposure to other common staining agents like tea or tobacco. Moreover, the study evaluated only short-term discoloration, limiting conclusions about long-term color stability. Future research should investigate various modeling liquids and staining protocols over extended periods to provide more comprehensive clinical insight. Therefore, the results of this study should be interpreted with caution and supported by further studies.

## Conclusions

Within the limitations of this study, it may be concluded that:

1. Modeling liquid application increased discoloration, depending on the composite type and whether polishing was performed. Higher ΔE₀₀ values were observed in the absence of polishing.
2. One-week coffee immersion led to clinically unacceptable ΔE₀₀ values in all one-shade composites, regardless of the surface treatment applied.
3. Glycerin gel application did not improve color stability when combined with modeling liquid and Mylar strip. It tended to increase ΔE₀₀ values, possibly due to air bubble entrapment and surface irregularities.

## Data Availability

The corresponding author can provide the data supporting the findings of this in vitro study upon request.
